# The Value of Indices and Measurements Used for Assessing Functional X-Rays of the Cervical Spine in Clinical Practice †

**DOI:** 10.3390/jcm14175995

**Published:** 2025-08-25

**Authors:** Marcin Janusz Łubiński, Piotr Kowalski, Filip Karol Kwiatkowski, Jolanta Ewa Kujawa, Gabriela Anna Figas, Agata Joanna Majos

**Affiliations:** 1 II Department of Radiology and Diagnostic Imaging, Central Teaching Hospital of the Medical University of Lodz, 92-213 Lodz, Polandagata.majos@umed.lodz.pl (A.J.M.); 2 Military Institute of Aviation Medicine, 01-755 Warsaw, Poland; 3 Medical Rehabilitation Clinic, Central Teaching Hospital of the Medical University of Lodz, 92-213 Lodz, Poland; jolanata.kujawa@umed.lodz.pl (J.E.K.); gabriela.figas@umed.lodz.pl (G.A.F.)

**Keywords:** cervical spine mobility, Cobb angle, extension, flexion, functional X-rays

## Abstract

**Background**: Functional X-ray imaging of the cervical spine in flexion and extension remains a fundamental, objective method for mobility assessment. However, there remains no consensus on how to perform the measurements or which indicators are most useful in clinical practice. **Methods**: This study included 288 participants (197 female and 91 male); these included patients without CDD signs or with first-degree cervical spine CDD according to the Kellgren–Lawrence criteria. Cobb angle C2–C7, HDI, ROM, CIF, and CIE were measured. **Results**: The most significant correlations were observed for HDI, and the strongest correlations were between CIF measurements. The greatest mobility was noted for the centrally located segments of the cervical spine, particularly at the C4–C5 level. **Conclusions**: HDI appears to be the most reliable parameter for characterizing the mobility of the cervical spine. It is precise and has the highest number of correlations with other measurements, but it is very time-consuming. Cobb angle C2–C7 combines ease of performance with good diagnostic value.

## 1. Introduction

Despite the passage of time and the dynamic development of diagnostic imaging, the most common method of assessing the cervical spine remains radiography—both in developing countries and in those with highly financed health care systems. While cervical spine mobility can be evaluated in various ways by clinicians and physiotherapists, imaging studies remain the most objective approach to assessing any changes. Functional tests performed in the flexion and extension positions provide important information [1,2,3,4]. These tests have high clinical value, as they allow the visualization and direct assessment of both the bone structures of the spine and, indirectly, the intervertebral discs. In many cases, disorders that cause clinical symptoms, such as headaches and dizziness, neck pain, numbness of the limbs, sensory disturbances, or limitation of motor skills, occur during movement and cannot be identified in static projections.

However, there is still a need for clear guidance on which radiological measurement or indicator fully illustrates the functional disorders of the cervical spine, thus enabling reliable diagnosis and characterization of pathological changes. Therefore, the purpose of the present study was to determine the most reliable and clinically useful method of measuring cervical spine mobility on functional X-rays.

## 2. Materials and Methods

### 2.1. Patients

This study involved a group of 288 participants (197 female and 91 male) aged 19–78 years; they were selected from the participants qualified for the VRneck SOLUTION project, a medical experiment entitled “Evaluation of the usefulness of available 3D technologies for visualization of functional motor tasks in the cervical spine”. Inclusion criteria included participants with and without neck pain and without cervical disc degeneration (CDD) or with its first degree diagnosed according to the Kellgren–Lawrence scale [5].

The following exclusion criteria were applied: kyphotic positioning of the cervical spine, congenital defects, post-surgical, post-traumatic and neoplastic lesions, or radiological features of osteoporosis and spinal instability, defined as horizontal displacement of adjacent vertebral bodies in functional projections by ≥4 mm [6,7]. Where the body of C6 was not included in the X-ray, the Cobb angle measurements were abandoned; however, other measurements were taken at the other levels included in the study.

The mean age of the study population was 44 years (±15.67): 45 years (±15.98) for women and 41 (±14.53) for men. The study participants were also stratified by age: 98 (63 F and 35 M) were between 19 and 30 years of age, 74 (48F, 26M) were 31–40 years, 68 (44F, 24M) were 41–50 years, 31 (26F, 5M) were 51–60 years, and 17 patients (16F, 1M) were aged over 60 years.

### 2.2. Methodology for Performing Functional X-Rays of the Cervical Spine

Each patient received three radiographs: one static lateral (L) and two functional, i.e., in flexion and extension. The radiographs showed the structures in their actual dimensions. All examinations were performed according to strict electro radiological technique standards [8]; detailed specifications are available in Appendix A.

The imaging was performed in three different centers in Poland; however, the images were assessed in the leading unit, i.e., the Central Teaching Hospital of the Medical University of Lodz. Each radiograph was assessed twice by two radiologists with several years of experience in assessing X-rays independently to check the reproducibility of the readings. Statistically significant differences between measurements were not observed.

### 2.3. Methods of Statistical Analysis

Discrete (categorical) variables were presented as integers (abundance) and percentages (frequency). Measurable (numeric) traits were described using the weighted arithmetic mean or median and by the standard deviation and minimum and maximum values.

The Shapiro–Wilk W-test was used to assess the normality of the distribution of the measurable trait, and Levene’s test was used to assess the homogeneity of variance. Variables with a normal distribution were analyzed using multivariate analysis of variance (ANOVA) without repetition, and those with a non-normal distribution were subjected to generalized multivariate linear models. Spearman’s rank correlation coefficient was calculated to estimate the relationship between selected numerical characteristics. To compare the frequencies of discrete variables, the χ^2^ test of independence, Fisher’s exact test for a small number of cells in the table (i < 5), or generalized linear models were used.

Values of *p* < 0.05 were considered statistically significant. All statistical calculations were performed using Statistica, version 14 (TIBCO Software Inc., Palo Alto, CA, USA).

Based on the collected data, mean and median values were determined for the following: Cobb C2–C7 angle in flexion and extension, segmental angular mobility, horizontal mobility index, and segmental cervical curvature, including both functional projections. Calculations were made for the general population, and according to the sex and specific age groups of the participants (viz. 19–30 years of age, 31–40, 41–50, 51–60, and 60+).

Where the changes in the functional projections were in the opposite direction than expected, they were assigned negative values.

A value of power analysis a priori was estimated at 0.9–0.95, and post hoc was >0.99.

### 2.4. Indicators and Measurements in Functional Examinations of the Cervical Spine

The following parameters were assessed in the functional X-ray radiographs of the cervical spine: the Cobb C2–C7 angle in the neutral position, flexion and extension, segmental angular and horizontal mobility, and indicators of segmental cervical curvature in flexion and extension.

### 2.5. Cobb Angle

**Figure 1:**
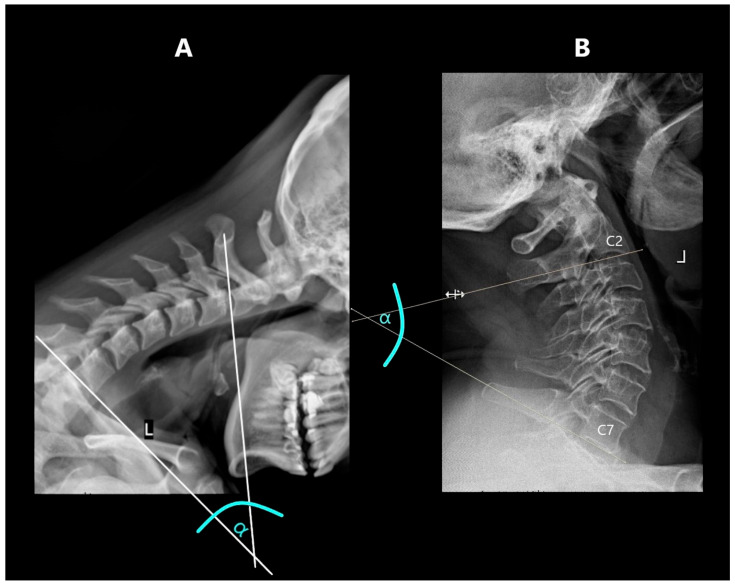
Cobb’s angle measurement: (**A**)—in flexion, (**B**)—in extension.

The Cobb angle was determined as the angle between two straight lines drawn tangentially to the lower edge of the C2 and C7 (Figure 1) [9,10]. Where the lower edge of C7 was not visible in the examination, due to the patient’s inability to take the required position, a C2–C6 substitute measurement with comparable diagnostic value was made [11]. Where the lower edge of the C6 vertebra was also unavailable, the measurements were abandoned (a total of 13 examinations in 11 patients). After taking measurements in functional projections, the sum of the obtained values was also calculated.

### 2.6. Horizontal Displacement Index (HDI)

**Figure 2:**
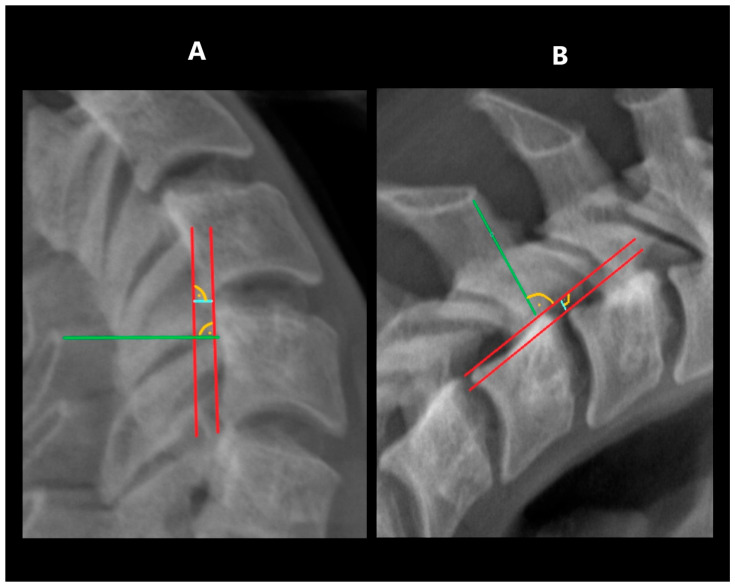
Measuring HDI: (**A**)—in extension, (**B**)—in flexion. Tangent lines to the posterior edge of vertebras—red lines; horizontal displacement distance—blue lines; spinal canal width—green lines.

The range of horizontal segmental mobility was measured in both functional projections. This was performed by measuring the displacement of the posterior edge of the upper vertebral body in relation to the posterior edge of the lower vertebral body. In flexion projection, the upper vertebral body should shift ventrally (forward), while in the extension projection, there should be a shift dorsally (backward). The values measured in flexion and extension were then related to the width of the spinal canal at the level of the lower vertebra. Measurements were taken at the narrowest point between the posterior contour of the vertebral body and the anterior contour of the spinous process of the same vertebra [6,12] (Figure 2).

### 2.7. Range of Angular Motion (ROM)

**Figure 3:**
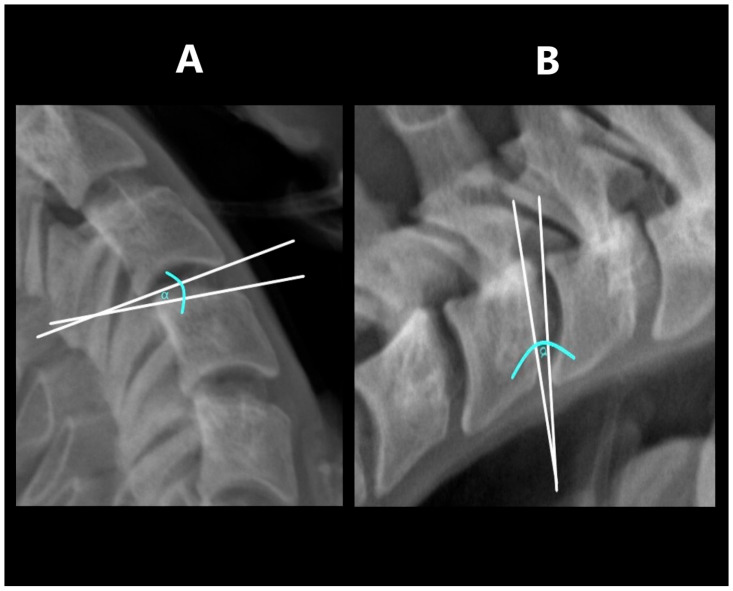
Measuring ROM: (**A**)—in extension, (**B**)—in flexion.

The range of angular motion was measured in the flexion and extension projections. The measurement was performed by determining the tangent to the lower contour of the upper vertebra and a second tangent drawn to the upper contour of the lower vertebra (Figure 3). The sum of the angles obtained in flexion and extension at a specific level was defined as its segmental angular mobility.

This study also determined the overall ROM of the C2–C7 cervical spine by summing the segmental angular mobility values for each level.

### 2.8. Cervical Segmental Curvature Indices (CIE—Curvature in Extension; CIF—Curvature in Flexion)

**Figure 4:**
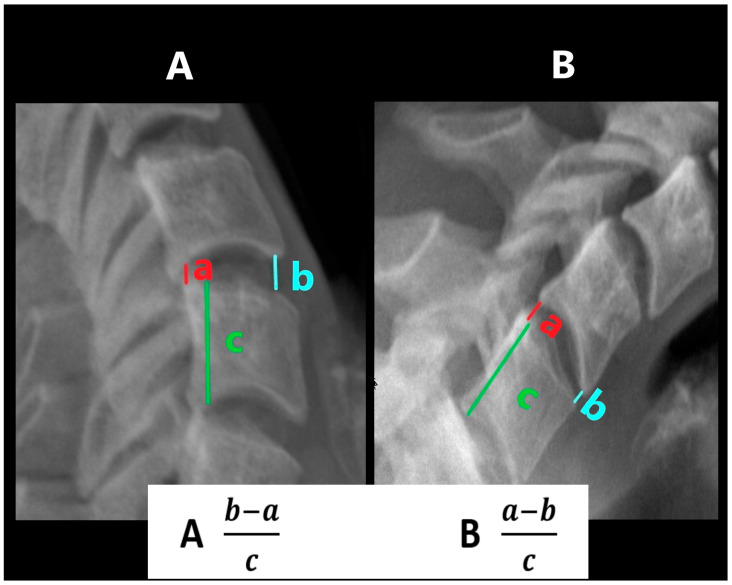
Measurement of curvature in extension (CIE) and in flexion (CIF). (**A**)—CIE, (**B**)—CIF.

The cervical segmental curvature index was defined as the difference between the heights in the anterior and posterior parts of a particular intervertebral space in relation to the height of the lower vertebral body, which was measured in its posterior part (Figure 4). In flexion, the difference between the sizes of the posterior and anterior parts was counted, while in extension, the size of the posterior part was subtracted from the size of the anterior part [6].

The cervical segmental curvature index was also measured for the entire C2–C7 spine by summing the indices of the individual constituent units (C2–C3, C3–C4, C4–C5, C5–C6 and C6–C7) in the flexion and extension position: total curvature in extension (tCIE); total curvature in flexion (tCIF).

## 3. Results

### 3.1. Cobb Angle at Rest

The mean Cobb’s angle at rest was 17.55°, the median was 16.20°, and the standard deviation (SD) was ±12.05°. The lowest value (0.10°) was observed in a woman in the 31–40 age group, and the highest (63.30°) in a 21-year-old man. The highest median (22.76°) and mean (21.80°) values were observed in the oldest age group (60+ years). These results gradually decreased inversely with age; the lowest mean values (15.05°) were noted in the 31–40 age group, and the lowest median values (14.70°) in the 19–30 age group, i.e., the youngest. The male participants tended to demonstrate higher median and mean values.

Significant differences in Cobb angle at rest were noted with regard to sex (*p* = 0.030) and age (*p* = 0.0001); however, no significant difference was observed among the entire group of participants.

### 3.2. Cobb Angle in Flexion

The mean Cobb angle in flexion in the general population was 24.75°, with a standard deviation of ±12.18°. The highest mean values were observed in the youngest group (32.91°); this value decreased with age, with the lowest value being observed in the 60+ group (15.93°). Slightly higher mean values were observed in men than in women. The lowest value (0.3°) was observed in patients in the 41–50 year and 51–60 year groups, while the highest minimum value (11.9°) was observed among the youngest patients (19–30 years). The highest value (63.7°) was observed in the 41–50 year age range, while the lowest maximum value (46.30°) was observed in the 60+ patient group.

Significant differences in Cobb angle in flexion values were observed between CDD patients and non-CDD patients (*p* < 0.0001) and between age groups (*p* < 0.001). However, our findings indicate that sex had no significant effect (*p* = 0.2839).

### 3.3. Cobb Angle in Extension

The mean Cobb angle in extension was 41.89° in the entire population of patients (standard deviation ± 12.80°). The median values, similar to the Cobb angle measured in the flexion position, were the highest in the youngest age group (47.75°), and the lowest among the oldest (37.55°). The highest mean values were again observed in the 19–30 age group (46.38°), while the lowest was in the 51–60 age group (37.58°). Higher median and mean values were observed among women than among men. The maximum Cobb angle in extension (77.2°) was measured in a 33-year-old man, and the smallest (8.7°) was in a female patient aged 25.

The Cobb angle in extension differed significantly with regard to the general population (*p* < 0.001), as well as with age (*p* < 0.001) and sex (*p* = 0.0005).

### 3.4. Horizontal Displacement Index (HDI)

At each level of the cervical spine, the lowest measured value was 0. Among all participants, the highest median and mean values were observed at the C4–C5 level (in both cases 0.19), and the lowest was at the C6–C7 level (mean 0.10 and median 0.11). In the oldest group, the highest mean value (0.17) was observed at the C3–C4 level, while the highest median (0.17) was noted for both the C3–C4 and C4–C5 levels. Women demonstrated higher or equal median and mean values compared to men. All the described relationships were statistically significant. However, at the C3–C4 level, while HDI differed significantly with regard to age (*p* = 0.0002), no significant difference was observed with regard to age (*p* = 0.1745).

### 3.5. Range of Angular Motion (ROM)

The greatest mobility of the cervical spine was observed at the C4–C5 level, reflected in its mean and median values, and its upper and lower ranges. In two age groups, the greatest results were measured at other levels of the cervical spine: at C5–C6 in the 31–40 year group and at C3–C4 in the 51–60 year group.

In the case of ROM of the entire cervical spine, understood as the sum of the results obtained at all levels, higher results were observed in women, and by far the highest value (164°) was measured in a patient from the youngest (19–30 year) group; this group also demonstrated the highest median and mean results. These values gradually decreased, reaching the lowest values in patients over 60 years of age. The lowest result (15.4°) was measured in men in the 41–50 year group.

### 3.6. Cervical Curvature in Flexion Index (CIF)

At almost all levels, the mean and median values were negative, indicating that the intervertebral space was higher in the anterior part than in the posterior part. The only exceptions to this rule were a positive mean value (0.0045), observed at C5–C6 in the 31–40 year group, and individual median values of 0 at C4–C5 (51–60 group), C5–C6 (19–30, 31–40 and 51–60 group), and C6–C7 (19–30, 41–50, 60+ and female group).

In the age groups 19–30 and 31–40, the highest obtained median and mean values were observed at C5–C6. In older patients, the highest values were recorded at C6–C7. The lowest value (−0.43) was observed at C3–C4, and the highest (0.28) at C5–C6.

### 3.7. Cervical Curvature in Extension Index (CIE)

In contrast to CIF, the CIE value was taken in the extension position, resulting in the positive mean and median values at all levels of all age groups (Table 1). However, each group had a few patient with negative CIE values.

The highest median and mean values were recorded at C4–C5 and C5–C6. Higher values were recorded at C5–C6 in the two youngest age groups and at C4–C5 in the remaining ones. The lowest median and mean results were recorded at C2–C3. Both the highest and the lowest results were observed at C2–C3.

## 4. Correlations

### 4.1. Cobb Angles

The Cobb angles measured at rest correlated positively with the Cobb angle in extension and negatively with the Cobb angle in flexion. The Cobb angle in flexion also correlated negatively with the Cobb angle in extension. The sum of the Cobb angles in the functional projections correlated with the ROM at each individual level and with the ROM of the entire spine.

At all levels, the Cobb angle at rest correlated with CIE. The Cobb angle in extension correlated with ROM and CIE at all levels; the Cobb angle in flexion correlated with all ROM values except ROM for C2–C3.

### 4.2. Horizontal Displacement Index (HDI)

All HDI measurements at each level showed positive correlations with the others. In addition, at all levels, the HDI measurements correlated positively with the Cobb angle in flexion and in extension; however, no correlation was noted between HDI and extension at C2–C3. In contrast, no correlations were observed between HDI and the Cobb angle at rest, except for a negative correlation at C2–C3. All HDI measurements at each level positively correlated with the ROM value at the same level. Correlations were also observed between the HDI values of the extreme spine levels (i.e., C2–C3 and C6–C7) and CIF and CIE of the corresponding levels. HDI correlated positively with CIF at C2–C3 and negatively at C6–C7; it also correlated negatively with CIE at C2–C3, C5–C6, and C6–C7.

### 4.3. Range of Angular Motion (ROM)

The ROM measurements of the levels demonstrated significant positive correlations with all other levels. In addition, positive correlations were observed between ROM and CIE values, with the exception of the two extremes of ROM (C2–C3 and C6–C7), where correlations occurred only at selected levels (ROM C2–C3 with CIE C2–C3, while ROM C6–C7 with CIE C5–C6 and C6–C7). Each ROM value also correlated with tCIE, except at C2–C3.

### 4.4. Cervical Curvature in Flexion Indices (CIF and CIE)

All CIF measurements correlated significantly with the CIF values of all other evaluated levels, as well as with tCIF. These were the strongest correlations in the study (Table 2). At the two lowest levels (C5–C6 and C6–C7), CIF correlated positively with most CIEs, while one negative correlation was found between CIF C5–C6 and CIE C2–C3. All CIE measurements correlated significantly with the CIE values of all evaluated levels, as well as with tCIE.

## 5. Discussion

With over 100 years of history of X-rays, many cervical spine mobility assessment methods have been proposed. In 1948, Cobb popularized the four-line method for measuring spinal curvature [13]. This method involved drawing two straight lines tangent to the vertebral endplates and then drawing two straight lines perpendicular to these lines. The angle formed at the intersection of these two lines was the Cobb angle. The measurement was then simplified by Drexler to a two-linear method with lower measurement risk error [14]. In 1977, Penning proposed a method for measuring spinal mobility by superimposing two films (the larger film in flexion is covered by a smaller one in extension) so that the vertebral bodies and spinous processes of one of the vertebrae were perfectly aligned [4]. Then the line was drawn along the edge of the overlying film on the underlying film. The same procedure was repeated for the vertebra located one level higher. The angle between these lines was the mobility of the segment. In 1986, Harrison presented another interesting method by drawing tangents to the posterior contours of the vertebral bodies [15]. The angles between the tangents determined the range of motion.

The present study attempted to identify reliable and clinically-useful indicators for the assessment of functional examinations of the cervical spine. It was decided to focus on the most common measurement method, i.e., the Cobb C2–C7 angle and a series of measurements assessed at each level of the cervical spine, proposed by Alizada et al. [6]. This allowed us to assess the mobility of the cervical spine at each level individually and as a whole. The data were acquired from a large group of patients.

The most mobile level of the cervical spine was found to be the C4–C5 segment: it demonstrated the highest mean and median ROM, as well as HDI and CIE values (Table 3). This finding, however, contradicts those of some previous studies, which attribute the greatest ROM to the C5–C6 segment [3,6,16,17,18]. Our study included by far the largest group of patients. Comparative analysis of study results is difficult due to incomplete information regarding the studied populations, such as their ethnicity. Considering the authors’ origin, it can be assumed that the majority of participants were Asian. In contrast, our study focused mostly on Europeans. One of the studies also used a specific subgroup of patients [18]. Another factor influencing the differences in results may be the use of different methods for assessing cervical spine mobility. Recently, lifestyle has changed significantly due to the rapid development of technology. Most of the mentioned studies were performed before smartphones and electronic devices became widely available, which could also explain the discrepancies between the results.

While the greatest mobility was noted in the C4–C5 segment, this was followed by the adjacent levels, i.e., C3–C4 and C5–C6, with the least mobility being observed in the marginal segments C2–C3 and C6–C7. This may result from the fact that the region C3–C6, i.e., around the center of the cervical spine, has the greatest activity. This would have a direct impact on the number of overloads and injuries in this area; these tend to accumulate with age, and indeed, the mean and median ROM values were found to gradually decrease in older age groups. Our data also indicate positive correlations between HDI and ROM at most levels of the cervical spine; however, no such relationships were demonstrated by Alizada et al. [6].

### 5.1. Cobb Angle

Higher Cobb angle values correspond to greater curvature of the spine. Our findings clearly indicate that in the static position, the highest lordosis values are found in the oldest group of patients. Many patients demonstrate shallowing of the natural curvature of the cervical spine due to excessive use of personal computers, cell phones, and tablets; this can even result in kyphotic positioning in younger patients. This dependence can lead to a disorder known as Tech Neck Syndrome or Text Neck Syndrome [19,20,21], characterized by disorders of normal lordosis, including kyphotic positioning of the cervical spine. This may result in back and shoulder pain and the premature development of degenerative spine disease [18,19].

The highest ROM values and Cobb angles in flexion and extension were observed among the 19 to 30-year age group, with a gradual decrease in range of motion being recorded in the older patient groups. This may be due to the fact that degenerative disease tends to deepen with age [20]. However, as our study included patients without cervical spine CDD, or with only first-degree CDD on the Kellgren–Lawrence classification, there must be other factors limiting the range of mobility of the cervical spine. These may include limited soft tissue elasticity or reduced function of the ligamentous apparatus, surrounding fascia, or intervertebral joints; this may develop with, or independently of, bony changes (Table 4).

It is also noteworthy that large individual variation was observed among the patients. The highest Cobb angle at rest was observed in a male patient from the 19–30 age group, and the lowest in a woman from the 31–40 age group. In flexion, the highest value was measured in a 46-year-old patient, and the lowest in a patient only three years younger. In addition, only a slight age difference was noted between the highest Cobb angle in extension (a 33-year-old man) and the lowest (a 25-year-old patient). Hence, it appears that the Cobb angle is characterized by significant variation between patients.

The results suggest that the mobility of the cervical spine depends on many individual factors. Age and related progressive changes in our bodies are only part of the elements that affect our range.

Lordosis is the natural curvature of the cervical spine. A physiological deepening can be seen during neck extension, and a more kyphotic profile is noted during flexion. A positive correlation was noted between the Cobb angle at rest and the values in extension; this could be due to the fact that in both cases, the observations concerned cervical lordosis. The negative correlations between the Cobb angle at rest and extension, and the Cobb angle in flexion noted in the present study, suggest that higher lordosis at rest and in extension is associated with lower kyphosis in flexion. In addition, greater kyphosis in flexion is associated with lower lordosis at rest and extension.

ROM positively correlated with both the Cobb angle in flexion and in extension. This was to be expected, as both parameters relate to the angular range of motion of the spine (Table 5). As such, the measurement of the Cobb angle in functional X-ray projections can allow for faster evaluation, due to the smaller number of measurements; hence, it is a desirable choice for diagnosis in daily clinical practice.

### 5.2. Horizontal Displacement Index (HDI)

For most levels, HDI showed positive correlations with Cobb’s angle in flexion and extension; however, no such correlations were observed with Cobb’s angle at rest.

The highest mean HDI values were observed at C4–C5, and the lowest at C6–C7; this was true for the entire study group, as well as for the age and sex subgroups. The fact that low values were noted at the last level of the cervical spine may result from the gradual change in the natural curvature of the spine from cervical lordosis to thoracic kyphosis; the thoracic spine is also characterized by a decidedly smaller range of motion than the cervical segment.

### 5.3. Cervical Segmental Curvature Indices and Range of Angular Motion (CIE, CIF, and ROM)

The ROM of an individual level of the cervical spine can be calculated in two ways. The first is to determine the tangent to the lower contour of the upper vertebra and the second tangent to the upper contour of the lower vertebra [21,22,23]. Alternatively, the tangent to the upper vertebra is determined in the same way, while the second tangent is determined based on the lower contour of the lower vertebra [6,7,24]. In both cases, the sum of the angles obtained at the intersection of the tangents in the flexion and extension position is defined as range of motion for a given level. Both methods have a cutoff value of 11° for normal range of motion, suggesting that only insignificant differences exist between the two [6,7,25]. The former method was used in the present study.

**Figure 5:**
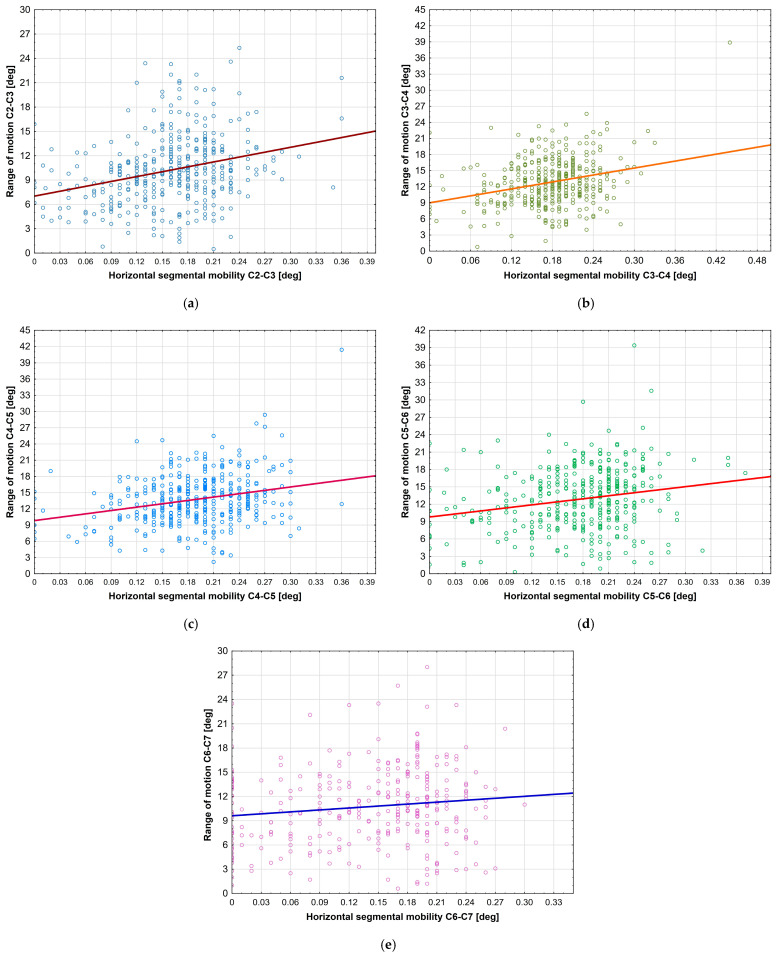
Correlations between HDI and ROM at (**a**) level C2–C3; (**b**) level C3–C4; (**c**) level C4–C5; (**d**) level C5–C6; (**e**) level C6–C7.

HDI and ROM were correlated at each level, suggesting that the displacement of the vertebral bodies in the horizontal plane is closely related to the change in the angle between them, which is extremely important clinically (Figure 5). This indicates that despite being divided into levels, the spine ultimately represents a physiological whole. It should be noted that Alizada et al. [6] reported no such relationship; however, the study used the second method of measuring ROM (i.e., based on the lower contour of the lower vertebra).

The female participants demonstrated slightly higher mean and median ROM for specific levels and for the entire cervical spine. They also exhibited a much wider range of maximum values at all levels compared to men.

ROM correlated with CIE at most levels, although no such correlations were observed with CIF. This may suggest that the position and shape of the intervertebral disc, and in the ligamentous apparatus, differ between positions. Considering that CIE also correlates with the Cobb angle at rest and flexion, this parameter appears to be a universal indicator in functional examinations of the cervical spine. In contrast, CIF does not correlate with practically any other indicators.

Interestingly, a significant number of CIF results were negative, as also noted by Alizada et al. [6]: negative values indicate a reverse displacement of the vertebral bodies relative to each other, compared to the expected value. In the case of CIF, the height in the posterior intervertebral space should be greater than in the anterior part. Our data may be influenced by inter alia increased tension of the neck extensor muscles, stiffness of the ligamentous apparatus, a sense of discomfort, or the influence of the flexion position on the organs of the neck, e.g., vessels.

## 6. Conclusions

In conclusion, HDI appears to be the most reliable parameter for characterizing the mobility of the cervical spine. It is precise and has the highest number of correlations with other measurements. As HDI needs to be assessed for each segment separately and requires a lot of precision, the measurement is time-consuming (takes up to 10–15 min). This can prove to be a significant barrier in daily clinical practice, when many patients need to be assessed in a relatively short time. Perhaps the rapidly developing artificial intelligence technology will help significantly speed up measurements. Thus, from a radiological perspective, the best indicator for assessing cervical spine mobility is arguably the Cobb C2–C7 angle, as it combines ease of performance with good diagnostic value. With the Cobb C2–C7 angle, a much larger group of patients can be reliably evaluated compared to HDI measurements, so it can also be used as a screening test to detect abnormalities, and, if present, HDI of every cervical spine segment can be assessed and used for follow-up assessment.

## Figures and Tables

**Table 1:** Descriptive statistics for segmental measurements of spine curvature.

Measurement	Section	Statistical Parameter *			
		M	SD	Me	Q_1_–Q_3_
Curvature in flexion (deg)	C2–C3	−0.0244	0.0783	−0.0300	(−0.0800)–0.0300
	C3–C4	−0.0263	0.0801	−0.0300	(−0.0800)–0.0300
	C4–C5	−0.0259	0.0724	−0.0230	(−0.0800)–0.0200
	C5–C6	−0.0077	0.0788	−0.0095	(−0.0600)–0.0400
	C6–C7	−0.0095	0.0857	0.0000	(−0.0700)–0.0400
	Overall	−0.1287	0.3937	−0.0900	(−0.3200)–0.0800
Curvature in extension (deg)	C2–C3	0.1116	0.0821	0.1100	0.0700–0.1500
	C3–C4	0.1543	0.0747	0.1500	0.1000–0.2000
	C4–C5	0.1724	0.0873	0.1700	0.1100–0.2300
	C5–C6	0.1717	0.0912	0.1700	0.1100–0.2300
	C6–C7	0.1483	0.0940	0.1500	0.0900–0.2000
	Overall	0.7009	0.4671	0.7000	0.5200–0.9000

* deg—degree, M—mean; SD—standard deviation; Me—median; Q—quartiles.

**Table 2:** Selected correlation coefficients for pairs of investigated spine measurements (the criterion for selecting the correlation coefficient |*r*| > 0.05).

Analyzed Variables *	*r*	*p*
CIF C4–C5 & tCIF	0.813	<0.0001
CIF C3–C4 & tCIF	0.782	<0.0001
CIF C5–C6 & CIF C6–C7	0.726	<0.0001
CIE C5–C6 & tCIE	0.725	<0.0001
CIF C5–C6 & tCIF	0.717	<0.0001
CIE C4–C5 & tCIE	0.716	<0.0001
CIF C6–C7 & tCIF	0.704	<0.0001
CIE C6–C7 & tCIE	0.703	<0.0001
CIF C2–C3 & tCIF	0.700	<0.0001
CIF C3–C4 & CIF C4–C5	0.687	<0.0001
Total Amount & ROM Total	0.687	<0.0001
Cobb angle in extension & Total Amount	0.651	<0.0001
HDI C3–C4 & HDI C4–C5	0.603	<0.0001
CIE C3–C4 & tCIE	0.601	<0.0001
Total Amount & ROM C5–C6	0.591	<0.0001
CIF C3–C4 & CIF C2–C3	0.580	<0.0001
CIF C2–C3 & CIF C3–C4	0.579	<0.0001
HDI C4–C5 & HDI C5–C6	0.579	<0.0001
CIE C5–C6 & CIE C6–C7	0.564	<0.0001
CIF C4–C5 & CIF C5–C6	0.560	<0.0001
CIE C2–C3 & tCIE	0.540	<0.0001
HDI C2–C3 & HDI C3–C4	0.538	<0.0001
CIE C4–C5 & CIE C5–C6	0.537	<0.0001
CIE C3–C4 & CIE C4–C5	0.530	<0.0001
Cobb angle in extension & ROM Total	0.530	<0.0001
CIF C2–C3 & CIF C4–C5	0.512	<0.0001
HDI C2–C3 & HDI C5–C6	0.505	<0.0001

* CIF—curvature in flexion, tCIF—total curvature in flexion, CIE—curvature in extension, tCIE—total curvature in extension, Total Amount—sum of Cobb angles in flexion and extension, ROM—range of angular motion.

**Table 3:** The highest mean and median values of individual indicators, considering cervical spine segments.

Indicator *	Mean		Median	
	Highest Value	2nd Highest Value	Highest Value	2nd Highest Value
HDI	0.1854 (C4–C5)	0.1751 (C3–C4)	0.1900 (C4–C5)	0.1800 (C3–C4, C5–C6)
ROM	13.7175 (C4–C5)	12.8370 (C3–C4)	13.4000 (C4–C5)	12.8000 (C5-C6)
CIF	−0.0077 (C5–C6)	−0.0095 (C6–C7)	0.0000 (C6–C7)	−0.0095 (C5–C6)
CIE	0.1724 (C4–C5)	0.1717 (C5–C6)	0.1700 (C4–C5, C5–C6)	0.1500 (C3–C4, C6–C7)

* HDI—horizontal displacement index, ROM—range of angular motion, CIF—curvature in flexion, CIE—curvature in extension.

**Table 4:** Descriptive statistics for Cobb angle measurements in the study cohort by age group.

Cobb Angle Measurement (deg)	Age Group (Years)	Statistical Parameter *			
		M	SD	Me	Q_1_–Q_3_
At rest	≤30	16.62	11.62	14.70	7.80–23.60
	31–40	15.05	10.43	15.20	5.00–22.00
	41–50	17.41	11.51	16.30	8.00–22.80
	51–60	20.28	11.16	19.40	11.00–26.90
	>60	22.76	13.58	21.80	13.50–32.10
	Overall	18.12	11.91	17.00	8.50–25.00
In extension	≤30	46.38	13.01	47.75	39.10–55.80
	31–40	41.64	12.21	38.85	34.30–50.00
	41–50	43.71	11.46	43.30	34.90–52.15
	51–60	37.58	12.43	39.20	28.20–46.40
	>60	37.76	12.94	37.55	28.40–46.00
	Overall	41.90	12.80	42.25	33.40–51.80
In flexion	≤30	32.91	10.38	32.40	25.10–39.00
	31–40	29.20	10.30	28.20	23.00–34.70
	41–50	22.45	10.98	21.30	16.00–29.50
	51–60	18.88	10.06	17.45	10.80–25.20
	>60	15.93	10.18	15.00	7.50–20.90
	Overall	24.75	12.18	24.90	16.10–32.30
Overall	≤30	78.82	15.37	79.40	35.80–116.80
	31–40	69.94	12.71	70.90	37.90–97.10
	41–50	62.32	17.87	64.20	0.00–89.60
	51–60	54.03	15.67	56.45	0.00–83.20
	>60	51.98	15.14	49.75	11.00–77.20
	Overall	64.71	18.45	65.55	0.00–116.80

* deg—degree, M—mean; SD—standard deviation; Me—median; Q—quartiles.

**Table 5:** Pearson correlation coefficients with their statistical significance for the range of motion (degree) and Cobb angle (degree) in the study cohort.

ROM (deg) *	C2–C3		C3–C4		C4–C5		C5–C6		C6–C7		Overall	
Cobb Angle (deg)	*r*	*p*	*r*	*p*	*r*	*p*	*r*	*p*	*r*	*p*	*r*	*p*
Rest	0.015	0.7722	−0.014	0.7808	−0.008	0.8688	0.084	0.0987	−0.022	0.6987	0.039	0.4377
Extension	0.183	0.0002	0.258	<0.0001	0.269	<0.0001	0.390	<0.0001	0.372	<0.0001	0.454	<0.0001
Flexion	0.070	0.1687	0.221	<0.0001	0.230	<0.0001	0.357	<0.0001	0.354	<0.0001	0.379	<0.0001
Overall	0.245	<0.0001	0.369	<0.0001	0.386	<0.0001	0.591	<0.0001	0.587	<0.0001	0.687	<0.0001

* deg—degree; ROM—range of angular motion.

## Data Availability

The measurement data used to support the findings of this study are available from the corresponding author upon request.

## Abbreviations

The following abbreviations are used in this manuscript:

CDD	Cervical Disc Degeneration
F	Female
M	Male
L	Lateral projection
HDI	Horizontal displacement index
ROM	Range of angular motion
CIF	Curvature index in flexion
CIE	Curvature index in extension
tCIE	Total curvature index in extension
tCIF	Total curvature index in flexion

## Appendix A

Standard protocol of functional cervical spine X-rays in flexion and extension:

- The patient is erect or sitting in a lateral position;
- Centering point directed at the C4 vertebra;
- Collimation limited to the cervical spine;
- Shoulders relaxed and lowered as much as possible;
- Exposure at full exhalation;
- In flexion: tuck the chin into the chest as far as possible;
- In extension: lift the chin as high as possible;
- Source to Image Distance (SID): 150–180 cm;
- Exposure: 70 kV; 0.7 mAs.

**Table A1:** Descriptive statistics for all parameters measured in the study cohort.

Analyzed Trait *	Mean	Median	Min.	Max.	SD
Age (y)	44.0300	44.0000	19.0000	78.0000	15.6700
HDI C2–C3	0.1581	0.1700	0.0000	0.3600	0.0629
HDI C3–C4	0.1751	0.1800	0.0000	0.4400	0.0594
HDI C4–C5	0.1854	0.1900	0.0000	0.3600	0.0612
HDI C5–C6	0.1674	0.1800	0.0000	0.3700	0.0717
HDI C6–C7	0.1078	0.1100	0.0000	0.3000	0.0899
Cobb angle at rest	18.1204	17.0000	0.1000	63.3000	11.9143
Cobb angle in extension	41.8977	42.2500	8.7000	77.2000	12.7966
Cobb angle in flexion	24.7537	24.9000	0.3000	63.7000	12.1787
Total Amount	64.7145	65.5500	0.0000	116.8000	18.4493
ROM C2–C3	10.2306	9.9000	0.5000	25.3000	4.2143
ROM C3–C4	12.8370	12.7000	0.8000	38.9000	4.3314
ROM C4–C5	13.7175	13.4000	2.2000	41.4000	4.5520
ROM C5–C6	12.8285	12.8000	0.3000	39.4000	5.3407
ROM C6–C7	10.7182	10.5000	0.6000	28.0000	4.8114
ROM Total	57.5529	57.7000	15.4000	164.6000	17.2300
CIF C2–C3	−0.0244	−0.0300	−0.2400	0.1700	0.0783
CIF C3–C4	−0.0263	−0.0300	−0.4300	0.2200	0.0801
CIF C4–C5	−0.0259	−0.0230	−0.2100	0.2700	0.0724
CIF C5–C6	−0.0077	−0.0095	−0.2500	0.2800	0.0788
CIF C6–C7	−0.0095	0.0000	−0.3400	0.2600	0.0857
tCIF	−0.1287	−0.0900	−3.0300	1.0900	0.3937
CIE C2–C3	0.1116	0.1100	−0.3200	0.8000	0.0821
CIE C3–C4	0.1543	0.0747	0.1500	−0.0900	0.5200
CIE C4–C5	0.1724	0.0873	0.1700	−0.1600	0.6200
CIE C5–C6	0.1717	0.0912	0.1700	−0.1500	0.5600
CIE C6–C7	0.1483	0.0940	0.1500	−0.1000	0.7000
tCIE	0.7009	0.4671	0.7000	−6.3200	2.3700

* HDI—horizontal displacement index, Total Amount—sum of Cobb angles in flexion and extension, ROM—range of angular motion, CIF—curvature in flexion, tCIF—total curvature in flexion, CIE—curvature in extension.

**Table A2:** Descriptive statistics for all parameters measured in the female cohort.

Analyzed Trait *	Mean	Median	Min.	Max.	SD
Age (years)	45.4028	46.0000	20.0000	78.0000	15.9765
HDI C2–C3	0.1618	0.1700	0.0000	0.3600	0.0644
HDI C3–C4	0.1766	0.1800	0.0000	0.4400	0.0591
HDI C4–C5	0.1887	0.1900	0.0000	0.3600	0.0608
HDI C5–C6	0.1724	0.1800	0.0000	0.3700	0.0700
HDI C6–C7	0.1172	0.1300	0.0000	0.3000	0.0857
Cobb angle at rest	17.5546	16.2000	0.1000	56.7000	12.0458
Cobb angle in extension	42.9723	43.0500	8.7000	70.1000	12.6453
Cobb angle in flexion	24.5554	24.5000	0.3000	63.7000	12.4533
Total Amount	66.1611	66.2000	0.0000	116.8000	17.6123
ROM C2–C3	10.5282	10.1000	0.5000	25.3000	4.2830
ROM C3–C4	13.1939	12.8000	1.9000	38.9000	4.5071
ROM C4/–C5	13.8849	13.5000	2.2000	41.4000	4.7485
ROM C5–C6	12.9124	12.8000	0.9000	39.4000	5.5858
ROM C6–C7	10.6576	10.4500	0.6000	28.0000	4.9081
ROM Total	59.3432	59.8000	24.0000	164.6000	17.4248
CIF C2–C3	−0.0254	−0.0300	−0.2300	0.1500	0.0797
CIF C3–C4	−0.0313	−0.0300	−0.4300	0.2200	0.0849
CIF C4–C5	−0.0266	−0.0200	−0.2100	0.2700	0.0731
CIF C5–C6	−0.0085	−0.0060	−0.2500	0.2800	0.0798
CIF C6–C7	−0.0089	0.0000	−0.2000	0.2600	0.0869
Total curvature in flexion	−0.1336	−0.0900	−2.2300	1.0900	0.3930
CIE C2–C3	0.1115	0.1000	−0.1400	0.8000	0.0829
CIE C3–C4	0.1546	0.1500	−0.0900	0.5200	0.0744
CIE C4–C5	0.1727	0.1700	−0.0600	0.6200	0.0900
CIE C5–C6	0.1738	0.1700	−0.0200	0.5600	0.0910
CIE C6–C7	0.1413	0.1300	−0.1000	0.7000	0.0935
Total curvature in extension	0.6996	0.7000	−6.3200	2.3700	0.5236

* HDI—horizontal displacement index, Total Amount—sum of Cobb angles in flexion and extension, ROM—range of angular motion, CIF—curvature in flexion, tCIF—total curvature in flexion, CIE—curvature in extension.

**Table A3:** Descriptive statistics for all parameters measured in the male cohort.

Analyzed Trait *	Mean	Median	Min.	Max.	SD
Age (years)	40.9120	40.0000	19.0000	74.0000	14.5264
HDI C2–C3	0.1498	0.1500	0.0000	0.3100	0.0588
HDI C3–C4	0.1716	0.1800	0.0000	0.3100	0.0602
HDI C4–C5	0.1780	0.1900	0.0000	0.3000	0.0618
HDI C5–C6	0.1562	0.1700	0.0000	0.3500	0.0744
HDI C6–C7	0.0866	0.0400	0.0000	0.2800	0.0956
Cobb angle at rest	19.4189	19.0000	0.3000	63.3000	11.5506
Cobb angle in extension	39.4083	40.4000	11.0000	77.2000	12.8509
Cobb angle in flexion	25.2214	25.3000	2.2000	63.0000	11.5435
Total Amount	61.4392	64.6000	0.0000	111.9000	19.9041
ROM C2–C3	9.5712	9.5000	0.8000	23.4000	3.9961
ROM C3–C4	12.0432	12.0000	0.8000	23.0000	3.8107
ROM C4–C5	13.3390	13.0000	4.3000	24.7000	4.0662
ROM C5–C6	12.6353	12.4000	0.3000	24.7000	4.7449
ROM C6–C7	10.9013	10.5000	1.0000	23.5000	4.5316
ROM Total	53.5712	53.9000	15.4000	91.5000	16.1564
CIF C2–C3	−0.0224	−0.0250	−0.2400	0.1700	0.0755
CIF C3–C4	−0.0151	−0.0200	−0.1700	0.1400	0.0671
CIF C4–C5	−0.0242	−0.0350	−0.1800	0.1700	0.0712
CIF C5–C6	−0.0059	−0.0100	−0.1800	0.2200	0.0766
CIF C6–C7	−0.0113	0.0000	−0.3400	0.1500	0.0820
Total curvature in flexion	−0.1177	−0.0800	−3.0300	0.5100	0.3967
CIE C2–C3	0.1119	0.1100	−0.3200	0.5000	0.0808
CIE C3–C4	0.1537	0.1500	−0.0500	0.3700	0.0757
CIE C4–C5	0.1716	0.1600	−0.1600	0.4100	0.0813
CIE C5–C6	0.1669	0.1700	−0.1500	0.4500	0.0921
CIE C6–C7	0.1673	0.1700	−0.0600	0.3500	0.0931
Total curvature in extension	0.7036	0.6900	−0.5700	1.5100	0.3089

* HDI—horizontal displacement index, Total Amount—sum of Cobb angles in flexion and extension, ROM—range of angular motion, CIF—curvature in flexion, tCIF—total curvature in flexion, CIE—curvature in extension.

**Table A4:** Descriptive statistics for all parameters measured in the 19–30-year-olds cohort.

Analyzed Trait *	Mean	Median	Min.	Max.	SD
HDI C2–C3	0.1778	0.1900	0.0000	0.3600	0.0589
HDI C3–C4	0.1915	0.1900	0.0000	0.4400	0.0603
HDI C4–C5	0.2005	0.2100	0.0000	0.3600	0.0590
HDI C5–C6	0.1914	0.2000	0.0000	0.3500	0.0560
HDI C6–C7	0.1286	0.1600	0.0000	0.2700	0.0844
Cobb angle at rest	16.6212	14.7000	0.4000	63.3000	11.6210
Cobb angle in extension	46.3776	47.7500	8.7000	71.0000	13.0114
Cobb angle in flexion	32.9152	32.4000	11.9000	63.0000	10.3844
Total Amount	78.8242	79.4000	35.8000	116.8000	15.3736
ROM C2–C3	10.4163	10.1000	1.9000	21.6000	4.4542
ROM C3–C4	13.9867	13.5000	2.8000	38.9000	4.8107
ROM C4–C5	14.5857	14.3500	4.0000	41.4000	5.0083
ROM C5–C6	15.8041	15.6500	5.9000	39.4000	5.0954
ROM C6–C7	13.0272	12.9000	4.3000	28.0000	4.3837
ROM Total	65.5602	64.5500	32.0000	164.6000	18.7503
CIF C2–C3	−0.0203	−0.0200	−0.2100	0.1400	0.0768
CIF C3–C4	−0.0169	−0.0300	−0.2000	0.1500	0.0628
CIF C4–C5	−0.0247	−0.0300	−0.1700	0.1500	0.0681
CIF C5–C6	−0.0003	0.0000	−0.1800	0.2200	0.0962
CIF C6–C7	−0.0035	0.0000	−0.3400	0.1800	0.1095
Total curvature in flexion	−0.0646	−0.0500	−0.9500	0.7200	0.3056
CIE C2–C3	0.0914	0.0900	−0.3200	0.3100	0.0800
CIE C3–C4	0.1524	0.1400	−0.0500	0.5200	0.0798
CIE C4–C5	0.1598	0.1500	−0.1600	0.6200	0.0915
CIE C5–C6	0.1879	0.1900	−0.0800	0.5600	0.0907
CIE C6–C7	0.1547	0.1500	−0.1000	0.3600	0.0871
Total curvature in extension	0.6453	0.6900	−6.3200	2.3700	0.7821

* HDI—horizontal displacement index, Total Amount—sum of Cobb angles in flexion and extension, ROM—range of angular motion, CIF—curvature in flexion, tCIF—total curvature in flexion, CIE—curvature in extension.

**Table A5:** Descriptive statistics for all parameters measured in the 31–40-year-olds cohort.

Analyzed Trait *	Mean	Median	Min.	Max.	SD
HDI C2–C3	0.1672	0.1750	0.0000	0.2900	0.0607
HDI C3–C4	0.1857	0.1900	0.0000	0.3100	0.0522
HDI C4–C5	0.2028	0.2000	0.0000	0.3000	0.0531
HDI C5–C6	0.1804	0.2000	0.0000	0.3500	0.0670
HDI C6–C7	0.1209	0.1500	0.0000	0.3000	0.0961
Cobb angle at rest	15.0519	15.2000	0.1000	50.3000	10.4323
Cobb angle in extension	41.6385	38.8500	14.5000	77.2000	12.2146
Cobb angle in flexion	29.1974	28.2000	5.7000	53.5000	10.3035
Total Amount	69.9392	70.9000	37.9000	97.1000	12.7135
ROM C2–C3	10.7795	10.2500	4.9000	25.3000	3.8457
ROM C3–C4	13.4180	13.2000	5.5000	25.6000	3.9466
ROM C4–C5	14.5128	14.1500	5.4000	27.2000	4.0678
ROM C5–C6	13.8909	14.2000	3.3000	29.7000	4.7242
ROM C6–C7	12.2309	11.8500	2.8000	25.7000	4.6594
ROM Total	63.0859	63.8000	37.6000	100.9000	14.2794
CIF C2–C3	−0.0117	−0.0200	−0.1700	0.1500	0.0680
CIF C3–C4	−0.0279	−0.0400	−0.2100	0.1500	0.0665
CIF C4–C5	−0.0161	−0.0110	−0.1800	0.1600	0.0620
CIF C5–C6	0.0045	0.0000	−0.1750	0.2000	0.0763
CIF C6–C7	−0.0242	−0.0200	−0.2000	0.1800	0.0869
Total curvature in flexion	−0.1400	−0.0500	−2.2300	0.5900	0.4481
CIE C2–C3	0.1103	0.1000	−0.0800	0.2700	0.0644
CIE C3–C4	0.1508	0.1500	0.0200	0.2600	0.0532
CIE C4–C5	0.1650	0.1600	0.0400	0.3700	0.0708
CIE C5–C6	0.1672	0.1700	−0.1500	0.3700	0.0867
CIE C6–C7	0.1559	0.1550	−0.0600	0.3000	0.0828
Total curvature in extension	0.7150	0.6900	−0.1900	1.2800	0.2687

* HDI—horizontal displacement index, Total Amount—sum of Cobb angles in flexion and extension, ROM—range of angular motion, CIF—curvature in flexion, tCIF—total curvature in flexion, CIE—curvature in extension.

**Table A6:** Descriptive statistics for all parameters measured in the 41–50-year-olds cohort.

Analyzed Trait *	Mean	Median	Min.	Max.	SD
HDI C2–C3	0.1589	0.1600	0.0000	0.3600	0.0599
HDI C3–C4	0.1710	0.1750	0.0000	0.3200	0.0637
HDI C4–C5	0.1800	0.1800	0.0000	0.3600	0.0592
HDI C5–C6	0.1643	0.1800	0.0000	0.3700	0.0733
HDI C6–C7	0.0983	0.1000	0.0000	0.2800	0.0863
Cobb angle at rest	17.4097	16.3000	0.3000	53.4000	11.5116
Cobb angle in extension	43.3076	43.3000	17.9000	70.0000	11.4648
Cobb angle in flexion	22.4506	21.3000	0.3000	63.7000	10.9759
Total Amount	62.3167	64.2000	0.0000	89.6000	17.8676
ROM C2–C3	9.8642	9.7000	1.4000	21.0000	4.0739
ROM C3–C4	12.8708	12.6000	0.8000	23.6000	4.2907
ROM C4–C5	14.1305	13.4000	4.3000	29.4000	4.6836
ROM C5–C6	13.1604	13.8000	3.2000	24.0000	4.5599
ROM C6–C7	10.5479	10.2000	0.6000	20.4000	4.4199
ROM Total	56.8917	56.7000	15.4000	103.4000	16.9121
CIF C2–C3	−0.0421	−0.0400	−0.2300	0.1400	0.0767
CIF C3–C4	−0.0297	−0.0350	−0.4300	0.2200	0.0962
CIF C4–C5	−0.0413	−0.0500	−0.2000	0.2700	0.0772
CIF C5–C6	−0.0077	−0.0100	−0.1700	0.2800	0.0722
CIF C6–C7	0.0011	0.0000	−0.1800	0.2600	0.0856
Total curvature in flexion	−0.1705	−0.1530	−3.0300	1.0900	0.4663
CIE C2–C3	0.1177	0.1200	−0.0900	0.5000	0.0790
CIE C3–C4	0.1575	0.1500	−0.0900	0.4000	0.0828
CIE C4–C5	0.1867	0.1900	−0.0600	0.3800	0.0862
CIE C5–C6	0.1751	0.1800	−0.0200	0.3600	0.0838
CIE C6–C7	0.1659	0.1600	0.0000	0.3900	0.0895
Total curvature in extension	0.7413	0.7650	−0.1700	1.3200	0.3004

* HDI—horizontal displacement index, Total Amount—sum of Cobb angles in flexion and extension, ROM—range of angular motion, CIF—curvature in flexion, tCIF—total curvature in flexion, CIE—curvature in extension.

**Table A7:** Descriptive statistics for all parameters measured in the 51–60-year-olds cohort.

Analyzed Trait *	Mean	Median	Min.	Max.	SD
HDI C2–C3	0.1436	0.1450	0.0000	0.2700	0.0642
HDI C3–C4	0.1589	0.1600	0.0000	0.2600	0.0488
HDI C4–C5	0.1721	0.1700	0.0000	0.3000	0.0593
HDI C5–C6	0.1508	0.1700	0.0000	0.3200	0.0761
HDI C6–C7	0.0947	0.0850	0.0000	0.2500	0.0943
Cobb angle at rest	20.2820	19.4000	1.6000	48.8000	11.1617
Cobb angle in extension	37.5833	39.2000	11.0000	60.7000	12.4317
Cobb angle in flexion	18.8776	17.4500	0.3000	46.6000	10.0628
Total Amount	54.0307	56.4500	0.0000	83.2000	15.6695
ROM C2–C3	10.3721	10.1000	0.5000	22.0000	3.8195
ROM C3–C4	12.3115	11.8000	5.5000	21.7000	3.5509
ROM C4–C5	12.6100	12.5500	2.2000	21.0000	3.7861
ROM C5–C6	10.2407	10.3000	1.6000	21.4000	4.8150
ROM C6–C7	8.5818	9.3000	1.0000	16.8000	3.9937
ROM Total	51.1820	52.3000	20.7000	73.9000	12.9125
CIF C2–C3	−0.0171	−0.0300	−0.1600	0.1300	0.0776
CIF C3–C4	−0.0261	−0.0300	−0.2100	0.1300	0.0795
CIF C4–C5	−0.0162	0.0000	−0.1900	0.1300	0.0726
CIF C5–C6	−0.0178	0.0000	−0.2100	0.1600	0.0684
CIF C6–C7	−0.0086	−0.0050	−0.1400	0.1200	0.0592
Total curvature in flexion	−0.1306	−0.1000	−2.0800	0.5000	0.3620
CIE C2–C3	0.1193	0.1000	−0.0600	0.8000	0.1131
CIE C3–C4	0.1597	0.1500	0.0300	0.3400	0.0754
CIE C4–C5	0.1684	0.1500	0.0000	0.4000	0.0948
CIE C5–C6	0.1575	0.1500	−0.1400	0.3500	0.0898
CIE C6–C7	0.1277	0.1050	−0.0600	0.7000	0.1160
Total curvature in extension	0.6970	0.6500	0.1200	1.9800	0.3249

* HDI—horizontal displacement index, Total Amount—sum of Cobb angles in flexion and extension, ROM—range of angular motion, CIF—curvature in flexion, tCIF—total curvature in flexion, CIE—curvature in extension.

**Table A8:** Descriptive statistics for all parameters measured in the 60+ cohort.

Analyzed Trait *	Mean	Median	Min.	Max.	SD
HDI C2–C3	0.1321	0.1400	0.0000	0.2500	0.0632
HDI C3–C4	0.1599	0.1700	0.0000	0.2700	0.0613
HDI C4–C5	0.1644	0.1700	0.0000	0.3100	0.0677
HDI C5–C6	0.1388	0.1450	0.0000	0.2900	0.0773
HDI C6–C7	0.0890	0.0800	0.0000	0.2700	0.0857
Cobb angle at rest	22.7643	21.8000	0.2000	56.7000	13.5796
Cobb angle in extension	37.7600	37.5500	13.7000	62.6000	12.9383
Cobb angle in flexion	15.9304	15.0000	2.0000	46.3000	10.1789
Total Amount	51.9778	49.7500	11.0000	77.2000	15.1436
ROM C2–C3	9.7329	9.0500	3.6000	23.4000	4.7627
ROM C3–C4	10.9914	10.6000	1.9000	18.4000	4.1371
ROM C4–C5	12.0043	11.4000	3.4000	22.2000	4.2649
ROM C5–C6	9.1382	9.3000	0.3000	21.4000	4.6751
ROM C6–C7	7.0420	7.0500	1.2000	19.8000	3.8716
ROM Total	46.6357	44.9000	24.0000	77.9000	13.6374
CIF C2–C3	−0.0265	−0.0400	−0.2400	0.1700	0.0909
CIF C3–C4	−0.0334	−0.0250	−0.3000	0.1400	0.0921
CIF C4–C5	−0.0262	−0.0200	−0.2100	0.1700	0.0803
CIF C5–C6	−0.0246	−0.0100	−0.2500	0.1500	0.0673
CIF C6–C7	−0.0149	0.0000	−0.1600	0.1400	0.0615
Total curvature in flexion	−0.1475	−0.1100	−1.9300	0.5100	0.3546
CIE C2–C3	0.1264	0.1400	−0.0800	0.3000	0.0708
CIE C3–C4	0.1519	0.1600	0.0000	0.3100	0.0772
CIE C4–C5	0.1820	0.1800	0.0000	0.4100	0.0914
CIE C5–C6	0.1616	0.1500	0.0000	0.5000	0.1062
CIE C6–C7	0.1221	0.1200	−0.0400	0.3900	0.0982
Total curvature in extension	0.7110	0.7200	−0.0400	1.7300	0.3254

* HDI—horizontal displacement index, Total Amount—sum of Cobb angles in flexion and extension, ROM—range of angular motion, CIF—curvature in flexion, tCIF—total curvature in flexion, CIE—curvature in extension.

**Table A9:** Correlation coefficients for all pairs of investigated spine measurements.

Analyzed Variables *	*r*	*p*
tCIF & CIF C4–C5	0.813	<0.0001
tCIF & CIF C3–C4	0.782	<0.0001
ROM Total & ROM C5–C6	0.743	<0.0001
CIF C5–C6 & CIF C6–C7	0.726	<0.0001
tCIE & CIE C5–C6	0.725	<0.0001
tCIF & CIF C5-C6	0.717	<0.0001
CIE C4-C5 & tCIE	0.716	<0.0001
ROM C6-C7 & ROM Total	0.712	<0.0001
CIF C6-C7 & tCIF	0.704	<0.0001
CIE C6-C7 & tCIE	0.703	<0.0001
CIF C2-C3 & tCIF	0.700	<0.0001
Total Amount & ROM Total	0.687	<0.0001
CIF C3-C4 & CIF C4-C5	0.687	<0.0001
ROM C3-C4 & ROM Total	0.681	<0.0001
ROM C4-C5 & ROM Total	0.665	<0.0001
Cobb angle in extension & Total Amount	0.651	<0.0001
HDI C3–C4 & HDI C4–C5	0.603	<0.0001
CIE C3–C4 & Total curvature in extension	0.601	<0.0001
Total Amount & ROM C5–C6	0.591	<0.0001
Total Amount & ROM C6–C7	0.587	<0.0001
Cobb angle in flexion & Total Amount	0.582	<0.0001
ROM C5–C6 & ROM C6–C7	0.580	<0.0001
CIF C2–C3 & CIF C3–C4	0.580	<0.0001
HDI C4–C5 & HDI C5–C6	0.579	<0.0001
CIE C5–C6 & CIE C6–C7	0.564	<0.0001
CIF C4–C5 & CIF C5–C6	0.560	<0.0001
Total Amount & CIE C3–C4	0.557	0.0341
CIE C2–C3 & tCIE	0.540	<0.0001
HDI C2–C3 & HDI C3–C4	0.538	<0.0001
CIE C4–C5 & CIE C5–C6	0.537	<0.0001
CIE C3–C4 & CIE C4–C5	0.530	<0.0001
Cobb angle in extension & ROM Total	0.530	<0.0001
ROM C2–C3 & ROM Total	0.513	<0.0001
CIF C2–C3 & CIF C4–C5	0.512	<0.0001
HDI C2–C3 & HDI C5–C6	0.505	<0.0001
ROM C4–C5 & ROM C5–C6	0.499	<0.0001
CIF C4–C5 & CIF C6–C7	0.493	<0.0001
ROM C3–C4 & ROM C4–C5	0.490	<0.0001
HDI C2–C3 & HDI C4–C5	0.483	<0.0001
HDI C3–C4 & HDI C5–C6	0.476	<0.0001
ROM C6–C7 & CIE C6–C7	0.473	<0.0001
HDI C5–C6 & HDI C6–C7	0.458	<0.0001
ROM C5–C6 & CIE C5–C6	0.451	<0.0001
CIF C3–C4 & CIF C5–C6	0.427	<0.0001
Cobb angle in extension & ROM C6–C7	0.423	<0.0001
Cobb angle in extension & ROM C5–C6	0.422	<0.0001
Total Amount & ROM C4–C5	0.386	<0.0001
CIE C4–C5 & CIE C6–C7	0.377	<0.0001
HDI C5–C6 & Total Amount	0.370	<0.0001
Total Amount & ROM C3–C4	0.369	<0.0001
ROM C3–C4 & ROM C6–C7	0.365	<0.0001
HDI C2–C3 & HDI C6–C7	0.364	<0.0001
ROM C3–C4 & ROM C5–C6	0.364	<0.0001
CIF C3–C4 & CIF C6–C7	0.354	<0.0001
ROM C4–C5 & ROM C6–C7	0.351	<0.0001
ROM C2–C3 & ROM C3–C4	0.348	<0.0001
CIE C3–C4 & CIE C5–C6	0.337	<0.0001
Cobb angle in flexion & ROM Total	0.336	<0.0001
Cobb angle in flexion & ROM C5–C6	0.334	<0.0001
Cobb angle at rest & Cobb angle in extension	0.324	<0.0001
ROM C5–C6 & tCIE	0.322	<0.0001
HDI C4–C5 & ROM Total	0.314	<0.0001
Cobb angle in flexion & ROM C6–C7	0.311	<0.0001
ROM Total & CIE C5–C6	0.309	<0.0001
ROM Total & tCIE	0.306	<0.0001
Cobb angle in extension & ROM C4–C5	0.306	<0.0001
CIE C2–C3 & CIE C3–C4	0.304	<0.0001
HDI C4–C5 & HDI C6–C7	0.302	<0.0001
HDI C5–C6 & ROM Total	0.301	<0.0001
ROM C5–C6 & CIE C6–C7	0.301	<0.0001
Cobb angle in extension & tCIE	0.300	<0.0001
Cobb angle in extension & ROM C3–C4	0.299	<0.0001
C4–C5 & Total Amount	0.294	<0.0001
ROM C4/C5 & CIE C4–C5	0.291	<0.0001
HDI C2–C3 & ROM C2–C3	0.289	<0.0001
HDI C6–C7 & Total Amount	0.287	<0.0001
ROM Total & CIE C6–C7	0.283	<0.0001
HDI C2–C3 & Total Amount	0.274	<0.0001
HDI C3–C4 & HDI C6–C7	0.270	<0.0001
Cobb angle in extension & CIE C5–C6	0.269	<0.0001
CIF C2–C3 & CIF C5–C6	0.268	<0.0001
HDI C5–C6 & Cobb angle in flexion	0.264	<0.0001
Cobb angle in extension & CIE C6–C7	0.264	<0.0001
ROM C3–C4 & CIE C3–C4	0.264	<0.0001
HDI C4–C5 & ROM C2–C3	0.263	<0.0001
HDI C6–C7 & ROM Total	0.259	<0.0001
CIE C2–C3 & CIE C4–C5	0.253	<0.0001
CIE C2–C3 & CIE C6–C7	0.251	<0.0001
HDI C3–C4 & Total Amount	0.250	<0.0001
Total Amount & CIE C6–C7	0.248	<0.0001
CIE C3–C4 & CIE C6–C7	0.248	<0.0001
HDI C4–C5 & ROM C3–C4	0.245	<0.0001
Total Amount & ROM C2–C3	0.245	<0.0001
HDI C5–C6 & ROM C2–C3	0.241	<0.0001
CIF C2–C3 & CIF C6–C7	0.241	0.0001
Cobb angle in extension & ROM C2–C3	0.238	<0.0001
ROM C4–C5 & tCIE	0.237	<0.0001
ROM C4–C5 & CIE C5–C6	0.235	<0.0001
ROM C6–C7 & CIE C5–C6	0.232	<0.0001
ROM C5–C6 & CIE C4–C5	0.228	<0.0001
CIE C2–C3 & CIE C5–C6	0.227	<0.0001
HDI C3–C4 & ROM C2–C3	0.226	<0.0001
ROM C2–C3 & CIF C2–C3	0.222	<0.0001
CIF C6–C7 & CIE C5–C6	0.220	0.0002
ROM C3–C4 & CIE C5–C6	0.216	<0.0001
HDI C4–C5 & ROM C4–C5	0.215	<0.0001
Cobb angle at rest & tCIE	0.211	<0.0001
HDI C3–C4 & ROM C3–C4	0.211	<0.0001
Cobb angle in flexion & HDI C2–C3	0.211	<0.0001
C2–C3 & Cobb angle in flexion	0.211	<0.0001
HDI C6–C7 & ROM C2–C3	0.210	<0.0001
ROM C2–C3 & ROM C4–C5	0.209	<0.0001
ROM C3–C4 & tCIE	0.209	<0.0001
HDI C5–C6 & ROM C6–C7	0.209	0.0002
CIF C6–C7 & CIE C4–C5	0.208	0.0005
HDI C5–C6 & ROM C5–C6	0.205	<0.0001
ROM C4–C5 & CIE C6–C7	0.204	0.0003
ROM C6–C7 & tCIE	0.204	0.0003
Cobb angle in flexion & ROM C4–C5	0.200	0.0001
HDI C2–C3 & ROM Total	0.199	0.0001
Total Amount & tCIE	0.199	0.0001
HDI C6–C7 & CIF C2–C3	0.195	0.0001
CIF C5–C6 & CIE C4–C5	0.195	0.0001
Cobb angle at rest & CIE C2–C3	0.194	0.0001
Total Amount & CIE C5–C6	0.192	0.0001
HDI C4–C5 & Cobb angle in flexion	0.190	0.0002
Cobb angle in flexion & C4–C5	0.190	0.0002
Cobb angle in extension & CIE C4–C5	0.187	0.0002
Cobb angle in flexion & ROM C3–C4	0.186	0.0002
ROM C5–C6 & CIF C6–C7	0.185	0.0020
HDI C3–C4 & ROM Total	0.184	0.0002
ROM C3–C4 & CIE C4–C5	0.181	0.0003
ROM C2–C3 & ROM C6–C7	0.180	0.0014
HDI C6–C7 & Cobb angle in flexion	0.178	0.0004
Cobb angle in flexion & HDI C6–C7	0.178	0.0004
CIF C6–C7 & tCIE	0.175	0.0036
ROM C2–C3 & ROM C5–C6	0.173	0.0006
ROM C5–C6 & CIF C5–C6	0.173	0.0006
ROM C2–C3 & CIE C2–C3	0.170	0.0006
HDI C4–C5 & ROM C6–C7	0.165	0.0033
ROM C3–C4 & CIE C6–C7	0.164	0.0034
ROM Total & CIE C4–C5	0.161	0.0012
ROM Total & CIE C3–C4	0.158	0.0015
CIF C5–C6 & CIE C6–C7	0.155	0.0057
ROM C5–C6 & CIE C3–C4	0.154	0.0023
ROM C4–C5 & CIE C3–C4	0.150	0.0027
Cobb angle in extension & CIE C2–C3	0.150	0.0029
CIF C5–C6 & CIE C5–C6	0.147	0.0039
Cobb angle in flexion & C3–C4	0.147	0.0036
HDI C3–C4 & Cobb angle in flexion	0.147	0.0036
HDI C4–C5 & ROM C5–C6	0.146	0.0038
HDI C5–C6 & CIF C2–C3	0.141	0.0046
HDI C3–C4 & ROM C6–C7	0.140	0.0129
Cobb angle at rest & CIE C6–C7	0.136	0.0160
ROM C4–C5 & CIF C5–C6	0.136	0.0075
CIF C6–C7 & CIE C6–C7	0.132	0.0306
Cobb angle at rest & CIE C5–C6	0.131	0.0095
Cobb angle at rest & CIE C3–C4	0.129	0.0102
Cobb angle in extension & CIE C3–C4	0.128	0.0106
Cobb angle in extension & HDI C3–C4	0.125	0.0124
HDI C3–C4 & Cobb angle in extension	0.125	0.0124
HDI C2–C3 & ROM C6–C7	0.123	0.0292
HDI C2–C3 & ROM C5–C6	0.121	0.0160
HDI C6–C7 & ROM C6–C7	0.120	0.0330
ROM Total & CIE C2–C3	0.119	0.0171
Cobb angle in extension & C4–C5	0.114	0.0228
HDI C4–C5 & Cobb angle in extension	0.114	0.0228
CIF C5–C6 & tCIE	0.114	0.0252
HDI C2–C3 & ROM C3–C4	0.112	0.0245
HDI C5–C6 & ROM C3–C4	0.112	0.0246
ROM C4–C5 & CIE C2–C3	0.112	0.0256
HDI C6–C7 & CIF C3–C4	0.111	0.0254
Cobb angle at rest & CIE C4–C5	0.109	0.0300
HDI C6–C7 & Cobb angle in extension	0.109	0.0297
Cobb angle in extension & HDI C6–C7	0.109	0.0297
HDI C5–C6 & Cobb angle in extension	0.108	0.0319
Cobb angle in extension & HDI C5–C6	0.108	0.0319
Cobb angle in flexion & CIF C5–C6	0.105	0.0392
ROM Total & CIF C5–C6	0.104	0.0404
HDI C2–C3 & CIF C2–C3	0.104	0.0374
ROM C5–C6 & CIE C2–C3	0.103	0.0408
ROM Total & CIF C2–C3	0.103	0.0392
ROM C3–C4 & CIF C4–C5	0.099	0.0484
ROM C2-C3 & CIF C3–C4	0.098	0.0490
HDI C2–C3 & CIE C2–C3	−0.102	0.0410
Cobb angle at rest & HDI C2–C3	−0.103	0.0399
HDI C2–C3 & Cobb angle at rest	−0.103	0.0399
CIF C5–C6 & CIE C2–C3	−0.103	0.0425
HDI C5–C6 & CIE C5–C6	−0.105	0.0357
HDI C5–C6 & tCIE	−0.111	0.0266
HDI C2–C3 & CIE C3–C4	−0.111	0.0258
Cobb angle in flexion & CIE C4–C5	−0.117	0.0216
HDI C2–C3 & CIE C5–C6	−0.120	0.0163
HDI C6–C7 & CIF C5–C6	−0.147	0.0037
HDI C2–C3 & CIF C6–C7	−0.147	0.0140
HDI C5–C6 & CIE C3–C4	−0.150	0.0026
HDI C5–C6 & CIE C6–C7	−0.156	0.0053
HDI C2–C3 & CIE C6–C7	−0.157	0.0051
Cobb angle in extension & Cobb angle in flexion	−0.157	0.0018
Cobb angle in flexion & CIE C2–C3	−0.173	0.0006
HDI C2–C3 & tCIE	−0.178	0.0003
HDI C6–C7 & CIE C6–C7	−0.186	0.0009
HDI C6–C7 & CIE C5–C6	−0.187	0.0002
HDI C5–C6 & CIE C4–C5	−0.198	0.0001
HDI C2–C3 & CIE C4–C5	−0.247	<0.0001
HDI C6–C7 & CIF C6–C7	−0.248	<0.0001
HDI C6–C7 & CIE C4–C5	−0.251	<0.0001
Cobb angle at rest & Cobb angle in flexion	−0.343	<0.0001

* CIF—curvature in flexion, tCIF—total curvature in flexion, CIE—curvature in extension, tCIE—total curvature in extension, Total Amount—sum of Cobb angles in flexion and extension, ROM—range of angular motion.
